# *N*-(4-Chloro­phenyl)-1,8-naphthalimide

**DOI:** 10.1107/S1600536809020777

**Published:** 2009-06-13

**Authors:** Sun Jie, Shuai Shao

**Affiliations:** a College of Food Science and Light Industry, Nanjing University of Technology, Xinmofan Road No.5 Nanjing, Nanjing 210009, People’s Republic of China

## Abstract

In the title compound, C_18_H_10_ClNO_2_, the naphthalimide ring system is almost planar, the rings forming dihedral angles of 2.05 (3), 2.26 (3) and 0.80 (3)°. The attached benzene ring of the 4-chloro­phenyl substituent is inclined to the mean plane of the naphthalimide ring system by 75.77 (11)°. In the crystal structure, symmetry-related mol­ecules are linked by C—H⋯O inter­actions. There are also weak π–π contacts between the naphthalimide rings [centroid–centroid distance = 3.732 (3) Å].

## Related literature

For related literature on *N*-substituted 1,8-naphthalimides, see: De Souza *et al.* (2002 ▶). For a description of the Cambridge Structural Database, see: Allen (2002 ▶). For hydrogen bonding, see: Bernstein *et al.* (1995 ▶).
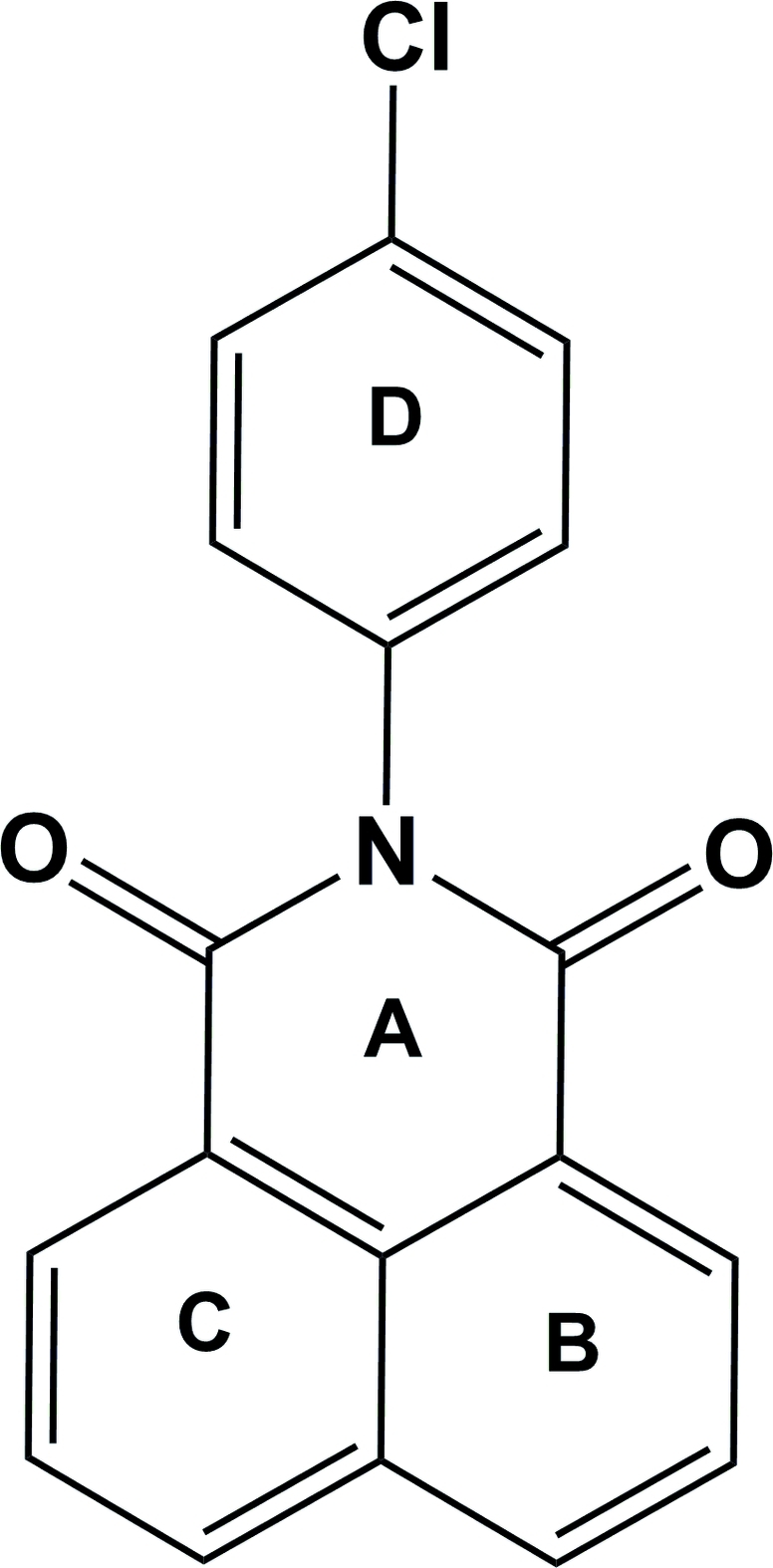


## Experimental

### 

#### Crystal data

C_18_H_10_ClNO_2_
*M* *_r_* = 307.72
Monoclinic, 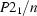

*a* = 8.6800 (17) Å
*b* = 17.553 (4) Å
*c* = 9.4600 (19) Å
β = 103.53 (3)°
*V* = 1401.3 (5) Å^3^
*Z* = 4
Mo *K*α radiation
μ = 0.28 mm^−1^
*T* = 293 K
0.30 × 0.20 × 0.20 mm

#### Data collection

Enraf–Nonius CAD-4 diffractometer
Absorption correction: ψ scan (North *et al.*, 1968 ▶) *T*_min_ = 0.921, *T*_max_ = 0.946
2719 measured reflections
2549 independent reflections
1843 reflections with *I* > 2σ(*I*)
*R*_int_ = 0.048
3 standard reflections every 200 reflections intensity decay: 1%

#### Refinement

*R*[*F* ^2^ > 2σ(*F* ^2^)] = 0.053
*wR*(*F* ^2^) = 0.157
*S* = 1.00
2549 reflections
199 parameters
H-atom parameters constrained
Δρ_max_ = 0.30 e Å^−3^
Δρ_min_ = −0.23 e Å^−3^

### 

Data collection: *CAD-4 EXPRESS* (Enraf–Nonius, 1989 ▶); cell refinement: *CAD-4 EXPRESS*; data reduction: *XCAD4* (Harms & Wocadlo, 1995 ▶); program(s) used to solve structure: *SHELXS97* (Sheldrick, 2008 ▶); program(s) used to refine structure: *SHELXL97* (Sheldrick, 2008 ▶); molecular graphics: *PLATON* (Spek, 2009 ▶); software used to prepare material for publication: *SHELXL97*.

## Supplementary Material

Crystal structure: contains datablocks global, I. DOI: 10.1107/S1600536809020777/su2116sup1.cif

Structure factors: contains datablocks I. DOI: 10.1107/S1600536809020777/su2116Isup2.hkl

Additional supplementary materials:  crystallographic information; 3D view; checkCIF report

## Figures and Tables

**Table 1:** Hydrogen-bond geometry (Å, °)

*D*—H⋯*A*	*D*—H	H⋯*A*	*D*⋯*A*	*D*—H⋯*A*
C15—H15*A*⋯O2^i^	0.93	2.45	3.138 (4)	131

Symmetry code: (i) 
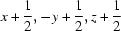
.

**Fig. 1.**
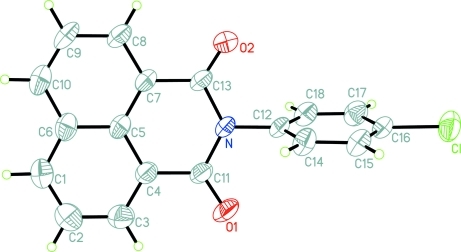
The molecular structure of the title compound, with the atom-numbering scheme. Displacement ellipsoids are drawn at the 50% probability level.

**Fig. 2.**
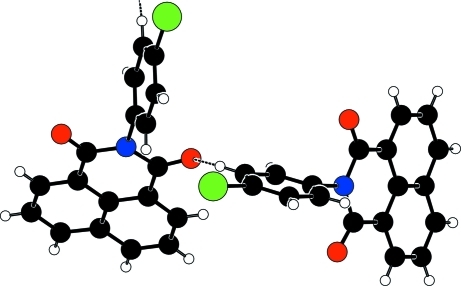
A view of the C-H···O hydrogen bonded (dashed lines) molecules in the title compound.

## supplementary crystallographic information

### Comment

As part of our ongoing studies on N-substituted 1,8-naphthalimides (De Souza et al., 2002), we report herein on the crystal structure of the title compound.

In the title compound, illustrated in Fig. 1, the bond lengths (Allen et al., 1987) and angles are within normal ranges. Rings A (N/C4/C5/C7/C11/C13), B (C1—C6) and C (C5—C11) are oriented with respect to one another by dihedral angles of A/B = 2.05 (3), A/C = 2.26 (3) and B/C = 0.80 (3) °, hence almost coplanar. Rings A, B (C5—C10), C (C9—C14) and D (C12/C14—C18) are oriented at dihedral angles of A/D = 76.89 (3), B/D = 75.93 (3) and C/D = 75.19 (3) °.

In the crystal structure, intermolecular C—H···O hydrogen bonds (Table 1) link the molecules into multimers (Fig. 2) (Bernstein et al., 1996), in which they may be effective in the stabilization of the structure. The π–π contacts between the naphthalimide rings, Cg1—Cg1^i^ [symmetry codes: (i) –X,-Y,-Z, where Cg1 is centroid of ring C] with centroid-centroid distances of 3.732 (3) Å, may further stabilize the structure.

### Experimental

For the preparation of the title compound: 1,8-naphthalic anhydride (1.98 g, 0.01 mol) and 2-aminoethanol (1.275 g,0.01 mol) were mixed with acetic acid (50 ml). The reaction mixture was refluxed for 8 h, and then poured into cold water. The resulting solids were filtered off and boiled with an aqueous solution of sodium bicarbonate (10%, 50 ml) for 20 min, and the insoluble solid residues were dried *in vacuo*. Column chromatography on aluminium oxide with benzene as eluent gave a light-brown solution. Crystals suitable for X-ray analysis were obtained by slow evaporation of an acetone solution (yield 94%; m.p. 489 K).

### Refinement

H-atoms were positioned geometrically and constrained to ride on their parent atoms: C—H = 0.93 Å with U_iso_(H) = 1.2U_eq_(parent C-atom).

### Crystal data

**Table:**

C_18_H_10_ClNO_2_	*F*(000) = 632
*M**_r_* = 307.72	*D*_x_ = 1.459 Mg m^−^^3^
Monoclinic, *P*2_1_/*n*	Melting point: 505 K
Hall symbol: -P 2yn	Mo *K*α radiation, λ = 0.71073 Å
*a* = 8.6800 (17) Å	Cell parameters from 25 reflections
*b* = 17.553 (4) Å	θ = 9–13°
*c* = 9.4600 (19) Å	µ = 0.28 mm^−^^1^
β = 103.53 (3)°	*T* = 293 K
*V* = 1401.3 (5) Å^3^	Block, green
*Z* = 4	0.30 × 0.20 × 0.20 mm

### Data collection

**Table:**

Enraf–Nonius CAD-4 diffractometer	1843 reflections with *I* > 2σ(*I*)
Radiation source: fine-focus sealed tube	*R*_int_ = 0.048
graphite	θ_max_ = 25.3°, θ_min_ = 2.3°
ω/2θ scans	*h* = 0→10
Absorption correction: ψ scan (North *et al.*, 1968)	*k* = 0→21
*T*_min_ = 0.921, *T*_max_ = 0.946	*l* = −11→11
2719 measured reflections	3 standard reflections every 200 reflections
2549 independent reflections	intensity decay: 1%

### Refinement

**Table:**

Refinement on *F*^2^	Primary atom site location: structure-invariant direct methods
Least-squares matrix: full	Secondary atom site location: difference Fourier map
*R*[*F*^2^ > 2σ(*F*^2^)] = 0.053	Hydrogen site location: inferred from neighbouring sites
*wR*(*F*^2^) = 0.157	H-atom parameters constrained
*S* = 1.00	*w* = 1/[σ^2^(*F*_o_^2^) + (0.07*P*)^2^ + 1.5*P*] where *P* = (*F*_o_^2^ + 2*F*_c_^2^)/3
2549 reflections	(Δ/σ)_max_ < 0.001
199 parameters	Δρ_max_ = 0.30 e Å^−^^3^
0 restraints	Δρ_min_ = −0.23 e Å^−^^3^

### Special details

**Table:**

Geometry. All e.s.d.'s (except the e.s.d. in the dihedral angle between two l.s. planes) are estimated using the full covariance matrix. The cell e.s.d.'s are taken into account individually in the estimation of e.s.d.'s in distances, angles and torsion angles; correlations between e.s.d.'s in cell parameters are only used when they are defined by crystal symmetry. An approximate (isotropic) treatment of cell e.s.d.'s is used for estimating e.s.d.'s involving l.s. planes.
Refinement. Refinement of *F*^2^ against ALL reflections. The weighted *R*-factor *wR* and goodness of fit *S* are based on *F*^2^, conventional *R*-factors *R* are based on *F*, with *F* set to zero for negative *F*^2^. The threshold expression of *F*^2^ > σ(*F*^2^) is used only for calculating *R*-factors(gt) *etc*. and is not relevant to the choice of reflections for refinement. *R*-factors based on *F*^2^ are statistically about twice as large as those based on *F*, and *R*- factors based on ALL data will be even larger.

### Fractional atomic coordinates and isotropic or equivalent isotropic displacement parameters (Å^2^)

**Table:**

	*x*	*y*	*z*	*U*_iso_*/*U*_eq_	
Cl	0.48706 (11)	0.18516 (6)	0.29044 (9)	0.0612 (3)	
N	0.6288 (3)	0.39484 (14)	−0.1550 (2)	0.0381 (6)	
O1	0.7353 (3)	0.48058 (14)	0.0210 (2)	0.0637 (7)	
C1	0.8795 (5)	0.5908 (2)	−0.4218 (4)	0.0606 (10)	
H1A	0.9182	0.6195	−0.4886	0.073*	
O2	0.5248 (3)	0.30743 (13)	−0.3284 (2)	0.0555 (6)	
C2	0.9122 (5)	0.6132 (2)	−0.2792 (4)	0.0705 (11)	
H2A	0.9710	0.6572	−0.2507	0.085*	
C3	0.8580 (4)	0.57040 (19)	−0.1762 (4)	0.0570 (9)	
H3A	0.8824	0.5856	−0.0794	0.068*	
C4	0.7693 (4)	0.50637 (17)	−0.2166 (3)	0.0413 (7)	
C5	0.7317 (4)	0.48249 (17)	−0.3649 (3)	0.0385 (7)	
C6	0.7888 (4)	0.52546 (18)	−0.4695 (3)	0.0459 (8)	
C7	0.6412 (3)	0.41605 (17)	−0.4090 (3)	0.0373 (7)	
C8	0.6044 (4)	0.3947 (2)	−0.5531 (3)	0.0498 (8)	
H8A	0.5435	0.3514	−0.5818	0.060*	
C9	0.6577 (4)	0.4375 (2)	−0.6567 (3)	0.0585 (10)	
H9A	0.6311	0.4227	−0.7538	0.070*	
C10	0.7487 (4)	0.5009 (2)	−0.6164 (4)	0.0541 (9)	
H10A	0.7849	0.5284	−0.6863	0.065*	
C11	0.7125 (4)	0.46182 (17)	−0.1071 (3)	0.0421 (7)	
C12	0.5915 (4)	0.34481 (17)	−0.0448 (3)	0.0376 (7)	
C13	0.5918 (4)	0.36798 (18)	−0.2992 (3)	0.0393 (7)	
C14	0.7103 (4)	0.30126 (18)	0.0375 (3)	0.0442 (8)	
H14A	0.8121	0.3040	0.0222	0.053*	
C15	0.6788 (4)	0.25313 (19)	0.1435 (3)	0.0464 (8)	
H15A	0.7592	0.2243	0.2014	0.056*	
C16	0.5257 (4)	0.24902 (17)	0.1611 (3)	0.0406 (7)	
C17	0.4056 (4)	0.29174 (19)	0.0787 (3)	0.0477 (8)	
H17A	0.3029	0.2875	0.0915	0.057*	
C18	0.4389 (4)	0.34117 (19)	−0.0239 (3)	0.0445 (7)	
H18A	0.3595	0.3718	−0.0785	0.053*	

### Atomic displacement parameters (Å^2^)

**Table:**

	*U*^11^	*U*^22^	*U*^33^	*U*^12^	*U*^13^	*U*^23^
Cl	0.0701 (6)	0.0711 (6)	0.0452 (5)	−0.0106 (5)	0.0189 (4)	0.0156 (4)
N	0.0516 (15)	0.0418 (14)	0.0203 (11)	−0.0043 (12)	0.0073 (10)	0.0008 (10)
O1	0.103 (2)	0.0626 (16)	0.0255 (11)	−0.0126 (14)	0.0140 (12)	−0.0104 (11)
C1	0.074 (2)	0.057 (2)	0.055 (2)	−0.0123 (19)	0.0224 (19)	0.0097 (17)
O2	0.0732 (16)	0.0572 (15)	0.0352 (12)	−0.0234 (13)	0.0107 (11)	−0.0085 (11)
C2	0.089 (3)	0.055 (2)	0.067 (3)	−0.022 (2)	0.017 (2)	−0.001 (2)
C3	0.075 (2)	0.047 (2)	0.0460 (19)	−0.0097 (18)	0.0081 (17)	−0.0038 (16)
C4	0.0537 (19)	0.0380 (17)	0.0310 (15)	−0.0019 (14)	0.0074 (13)	0.0002 (12)
C5	0.0443 (16)	0.0425 (17)	0.0282 (14)	0.0057 (13)	0.0073 (12)	0.0060 (12)
C6	0.0541 (19)	0.0453 (18)	0.0392 (17)	0.0081 (15)	0.0128 (14)	0.0128 (14)
C7	0.0448 (16)	0.0425 (17)	0.0244 (14)	0.0038 (13)	0.0079 (12)	0.0004 (12)
C8	0.066 (2)	0.057 (2)	0.0255 (15)	−0.0026 (17)	0.0088 (14)	−0.0026 (14)
C9	0.077 (3)	0.078 (3)	0.0226 (15)	0.002 (2)	0.0153 (15)	−0.0007 (16)
C10	0.066 (2)	0.061 (2)	0.0386 (17)	0.0044 (18)	0.0202 (16)	0.0125 (16)
C11	0.0581 (19)	0.0406 (17)	0.0260 (14)	−0.0014 (14)	0.0065 (13)	−0.0029 (12)
C12	0.0516 (18)	0.0420 (16)	0.0199 (13)	−0.0037 (14)	0.0099 (12)	−0.0026 (11)
C13	0.0484 (17)	0.0465 (18)	0.0219 (13)	−0.0011 (15)	0.0057 (12)	−0.0026 (12)
C14	0.0404 (16)	0.059 (2)	0.0346 (16)	0.0050 (15)	0.0117 (13)	0.0071 (14)
C15	0.0506 (19)	0.056 (2)	0.0323 (15)	0.0046 (15)	0.0080 (14)	0.0054 (14)
C16	0.0516 (18)	0.0461 (17)	0.0243 (14)	−0.0066 (14)	0.0094 (13)	−0.0001 (12)
C17	0.0428 (17)	0.064 (2)	0.0399 (17)	−0.0018 (16)	0.0164 (14)	−0.0010 (15)
C18	0.0435 (17)	0.0544 (19)	0.0346 (16)	0.0051 (14)	0.0070 (13)	0.0025 (14)

### Geometric parameters (Å, °)

**Table:**

Cl—C16	1.749 (3)	C7—C8	1.377 (4)
N—C11	1.401 (4)	C7—C13	1.477 (4)
N—C13	1.408 (3)	C8—C9	1.396 (5)
N—C12	1.457 (3)	C8—H8A	0.9300
O1—C11	1.226 (3)	C9—C10	1.367 (5)
C1—C2	1.370 (5)	C9—H9A	0.9300
C1—C6	1.404 (5)	C10—H10A	0.9300
C1—H1A	0.9300	C12—C14	1.370 (4)
O2—C13	1.212 (3)	C12—C18	1.386 (4)
C2—C3	1.396 (5)	C14—C15	1.386 (4)
C2—H2A	0.9300	C14—H14A	0.9300
C3—C4	1.365 (4)	C15—C16	1.379 (4)
C3—H3A	0.9300	C15—H15A	0.9300
C4—C5	1.427 (4)	C16—C17	1.370 (4)
C4—C11	1.472 (4)	C17—C18	1.382 (4)
C5—C7	1.414 (4)	C17—H17A	0.9300
C5—C6	1.423 (4)	C18—H18A	0.9300
C6—C10	1.418 (5)		
			
C11—N—C13	125.3 (2)	C8—C9—H9A	119.8
C11—N—C12	117.4 (2)	C9—C10—C6	120.9 (3)
C13—N—C12	117.0 (2)	C9—C10—H10A	119.5
C2—C1—C6	121.5 (3)	C6—C10—H10A	119.5
C2—C1—H1A	119.2	O1—C11—N	119.8 (3)
C6—C1—H1A	119.2	O1—C11—C4	123.3 (3)
C1—C2—C3	120.4 (4)	N—C11—C4	116.9 (2)
C1—C2—H2A	119.8	C14—C12—C18	120.7 (3)
C3—C2—H2A	119.8	C14—C12—N	118.6 (3)
C4—C3—C2	120.5 (3)	C18—C12—N	120.7 (3)
C4—C3—H3A	119.7	O2—C13—N	120.2 (3)
C2—C3—H3A	119.7	O2—C13—C7	122.9 (3)
C3—C4—C5	120.1 (3)	N—C13—C7	116.9 (3)
C3—C4—C11	120.0 (3)	C12—C14—C15	120.1 (3)
C5—C4—C11	119.9 (3)	C12—C14—H14A	120.0
C7—C5—C6	119.5 (3)	C15—C14—H14A	120.0
C7—C5—C4	121.1 (3)	C16—C15—C14	118.5 (3)
C6—C5—C4	119.4 (3)	C16—C15—H15A	120.7
C1—C6—C10	123.6 (3)	C14—C15—H15A	120.7
C1—C6—C5	118.0 (3)	C17—C16—C15	121.9 (3)
C10—C6—C5	118.4 (3)	C17—C16—Cl	120.3 (2)
C8—C7—C5	120.0 (3)	C15—C16—Cl	117.8 (2)
C8—C7—C13	120.2 (3)	C16—C17—C18	119.1 (3)
C5—C7—C13	119.8 (2)	C16—C17—H17A	120.4
C7—C8—C9	120.7 (3)	C18—C17—H17A	120.4
C7—C8—H8A	119.7	C17—C18—C12	119.6 (3)
C9—C8—H8A	119.7	C17—C18—H18A	120.2
C10—C9—C8	120.5 (3)	C12—C18—H18A	120.2
C10—C9—H9A	119.8		
			
C6—C1—C2—C3	1.1 (6)	C12—N—C11—C4	171.5 (3)
C1—C2—C3—C4	−1.1 (6)	C3—C4—C11—O1	2.9 (5)
C2—C3—C4—C5	0.2 (5)	C5—C4—C11—O1	−177.2 (3)
C2—C3—C4—C11	−179.9 (4)	C3—C4—C11—N	−177.0 (3)
C3—C4—C5—C7	179.5 (3)	C5—C4—C11—N	2.9 (4)
C11—C4—C5—C7	−0.4 (4)	C11—N—C12—C14	−75.1 (4)
C3—C4—C5—C6	0.7 (5)	C13—N—C12—C14	98.6 (3)
C11—C4—C5—C6	−179.3 (3)	C11—N—C12—C18	105.1 (3)
C2—C1—C6—C10	178.5 (4)	C13—N—C12—C18	−81.2 (4)
C2—C1—C6—C5	−0.3 (5)	C11—N—C13—O2	176.7 (3)
C7—C5—C6—C1	−179.5 (3)	C12—N—C13—O2	3.6 (4)
C4—C5—C6—C1	−0.6 (5)	C11—N—C13—C7	−2.1 (4)
C7—C5—C6—C10	1.7 (4)	C12—N—C13—C7	−175.3 (3)
C4—C5—C6—C10	−179.5 (3)	C8—C7—C13—O2	3.4 (5)
C6—C5—C7—C8	−2.1 (4)	C5—C7—C13—O2	−174.2 (3)
C4—C5—C7—C8	179.1 (3)	C8—C7—C13—N	−177.8 (3)
C6—C5—C7—C13	175.4 (3)	C5—C7—C13—N	4.6 (4)
C4—C5—C7—C13	−3.4 (4)	C18—C12—C14—C15	−0.3 (5)
C5—C7—C8—C9	1.0 (5)	N—C12—C14—C15	179.9 (3)
C13—C7—C8—C9	−176.5 (3)	C12—C14—C15—C16	1.5 (5)
C7—C8—C9—C10	0.6 (5)	C14—C15—C16—C17	−0.9 (5)
C8—C9—C10—C6	−1.1 (5)	C14—C15—C16—Cl	177.5 (2)
C1—C6—C10—C9	−178.9 (3)	C15—C16—C17—C18	−0.9 (5)
C5—C6—C10—C9	−0.1 (5)	Cl—C16—C17—C18	−179.3 (2)
C13—N—C11—O1	178.5 (3)	C16—C17—C18—C12	2.1 (5)
C12—N—C11—O1	−8.4 (4)	C14—C12—C18—C17	−1.5 (5)
C13—N—C11—C4	−1.6 (4)	N—C12—C18—C17	178.2 (3)

### Hydrogen-bond geometry (Å, °)

**Table:**

*D*—H···*A*	*D*—H	H···*A*	*D*···*A*	*D*—H···*A*
C15—H15A···O2^i^	0.93	2.45	3.138 (4)	131

Symmetry codes: (i) *x*+1/2, −*y*+1/2, *z*+1/2.

## References

[bb1] Allen, F. H. (2002). *Acta Cryst.* B**58**, 380–388. 10.1107/s0108768102003890 12037359

[bb2] Bernstein, J., Davis, R. E., Shimoni, L. & Chang, N.-L. (1995). *Angew. Chem. Int. Ed. Engl.* **34**, 1555–1573.

[bb3] De Souza, M. M., Correa, R., Cechinel Filho, V., Grabchev, I. & Bojinov, V. (2002). *Pharmazie*, **57**, 430-431. 12116885

[bb4] Enraf–Nonius (1989). *CAD-4 Software* Enraf–Nonius, Delft, The Netherlands.

[bb5] Harms, K. & Wocadlo, S. (1995). *XCAD4* University of Marburg, Germany.

[bb6] North, A. C. T., Phillips, D. C. & Mathews, F. S. (1968). *Acta Cryst.* A**24**, 351–359.

[bb7] Sheldrick, G. M. (2008). *Acta Cryst.* A**64**, 112–122. 10.1107/S0108767307043930 18156677

[bb8] Spek, A. L. (2009). *Acta Cryst.* D**65**, 148–155. 10.1107/S090744490804362X PMC2631630 19171970

